# Response of *Medicago truncatula* Seedlings to Colonization by *Salmonella enterica* and *Escherichia coli* O157:H7

**DOI:** 10.1371/journal.pone.0087970

**Published:** 2014-02-14

**Authors:** Dhileepkumar Jayaraman, Oswaldo Valdés-López, Charles W. Kaspar, Jean-Michel Ané

**Affiliations:** 1 Department of Agronomy, University of Wisconsin–Madison, Madison Madison, Wisconsin, United States of America; 2 Department of Bacteriology, University of Wisconsin–Madison, Madison, Wisconsin, United States of America; Leibniz-Institute for Vegetable and Ornamental Plants, Germany

## Abstract

Disease outbreaks due to the consumption of legume seedlings contaminated with human enteric bacterial pathogens like *Escherichia coli* O157:H7 and *Salmonella enterica* are reported every year. Besides contaminations occurring during food processing, pathogens present on the surface or interior of plant tissues are also responsible for such outbreaks. In the present study, surface and internal colonization of *Medicago truncatula,* a close relative of alfalfa, by *Salmonella enterica* and *Escherichia coli* O157:H7 were observed even with inoculum levels as low as two bacteria per plant. Furthermore, expression analyses revealed that approximately 30% of *Medicago truncatula* genes were commonly regulated in response to both of these enteric pathogens. This study highlights that very low inoculum doses trigger responses from the host plant and that both of these human enteric pathogens may in part use similar mechanisms to colonize legume seedlings.

## Introduction

The demand for fresh produce is greater than ever due to the changing lifestyle of people in industrialized nations [1]. In the United States alone, a 25% average increase in the per capita consumption of fresh produce was observed over the last two decades [2], [3]. The consumption of raw produce has led to an increase in the number of reported illnesses due to contaminated produce [4]. It is estimated that food-borne diseases cost $14.1 billion annually in the U.S. alone [5]. Between 1970 and 1990, raw produce-related outbreaks in the U.S. increased from 0.7% to 6.0%. Viruses were responsible for 20% of food-borne illnesses in which the agent was identified, and parasites were responsible for an additional 16% of infections [4]. Bacteria, however, were responsible for roughly 60% of the outbreaks that occurred between 2008 and 2011 and *Salmonella enterica* alone accounted for more than half of these bacterial outbreaks [3], [4]. Contaminated legume (alfalfa, clover, and bean) seedlings are frequently the cause of food-borne diseases from *Salmonella enterica* subsp. *enterica* (hereafter *S. enterica*) and *Escherichia coli* serovar O157:H7 (hereafter *E. coli* O157:H7) [6], [7].

Several factors have contributed to the increase in food-borne illness, one being an increase in contamination rates [1]. Even though seeds are surface-decontaminated during sprout production, contamination can occur during the pre- or post-harvest phases. Post-harvest contamination can occur from errors in food handling during washing, storage, rinsing, cutting, processing, or consumption [8]. Pathogenic bacteria such as *S. enterica* show a specific tropism towards cut surfaces and target cuts in leaves [9]. Mechanical damage can also distribute pathogenic bacteria on produce, leading to the contamination of edible parts. For example, melons left at room temperature can become contaminated when, upon cutting, bacterial pathogens present on the rind are transferred to the edible flesh [10]. In addition to contamination during food handling, recent evidence suggests that pre-harvest contamination also occurs by seed infection and bacterial colonization prior to germination. This colonization may occur in the leaf, shoots, or root tissues [11].

A wide spectrum of microbes colonizes the phyllosphere, and the degree of colonization is governed by the phenotype of the host plant, the water and phosphorus content of the leaf, the leaf thickness, and the quantity of bacterial growth inhibiting phenolics in the leaf [12]. Generally, the phyllosphere is considered a hostile environment for colonization by enteric bacteria due to the limited supply of water and nutrients and highly fluctuating environmental conditions [13], [14].

The enteric pathogens *S. enterica* and *E. coli* O157:H7 preferentially colonize root versus shoot tissues [8], [13]. Root exudates are rich in metabolites, which play a role in attracting, selecting and feeding microbes in the rhizosphere [15], [16]. The entry of microbes into the plant through cracks on the root surface is a common phenomenon [17]. Cracks generally open at the point of emergence of lateral roots from the primary root, a region where these enteric bacteria are found in high doses. This region is also abundant in root exudates [13], [17]. These root exudates contain carbon sources that enable the bacteria to proliferate in the rhizosphere and colonize the root surface [18], [19]. These carbon sources may be involved in the chemotaxic movement of bacterial cells towards the roots [18], [20]. Despite widespread occurrences of food-borne illness due to raw produce consumption, very little is known about the genetics governing the colonization of bacteria in the plant tissues. Whether this process is controlled at the host level and/or is governed by the pathogens remains controversial.

In the present work, we have selected *Medicago truncatula* (hereafter Medicago) as a model for studying the interactions with enteric bacteria *S. enterica* and *E. coli* O157:H7. This plant is closely related to alfalfa (*Medicago sativa*) and forms nitrogen-fixing associations with soil bacteria called rhizobia [21]–[23]. The annual habitat of Medicago, as well as its relatively short life cycle, its diploid and self-fertile nature with abundant natural variation, and its close phylogenetic relationship with many crop legumes, makes it an excellent model plant. Besides these natural attributes, various tools have been developed for this plant, including an efficient transformation system [24], [25], insertional mutagenesis [26], [27], RNA interference (RNAi) [28], virus-induced gene silencing (VIGS) [29], [30] and well-characterized cytogenetics [31], [32]. Recently, gene-rich regions of Medicago have been sequenced, enabling the prediction of 62,388 gene loci with 14,322 of these loci annotated as transposons [33]. The availability of these tools makes Medicago a unique system for studying the genetics of host plant infections.

Seedlings (e.g. alfalfa) are one source of salmonellosis [6]. The cool and dry conditions used for the storage of alfalfa seeds provide an ambient environment for the survival of *S. enterica*. During the sprouting process, which lasts 4–12 days, a rapid increase in *S. enterica* populations can occur and probably does not decrease during subsequent refrigeration. From farm to table, many opportunities exist for the contamination of alfalfa seeds or seedlings. Although various approaches have been developed for the surface sterilization of seeds, none are foolproof [34]–[36]. This shortcoming is relevant considering that even relatively low levels of surviving pathogens (<0.1 colony forming units (CFU)/g) can grow to densities in excess of 1×10^6 ^CFU/g only 48 hours into the sprouting process [37]. Additionally, enteric pathogens may colonize the internal tissues of the plant and escape surface sterilization [17], [38], [39]. Several groups have reported the penetration of enteric pathogens inside plant tissues. However, these findings remain controversial either because high doses of the initial inoculum were used or the method of inoculation used causes mechanical damage to the plants.

The present study was conducted with the goals of: (i) testing if and how the plant responds to human enteric pathogens at low inoculums levels and (ii) testing the similarities between these responses. Our results indicate that the enteric pathogens *S. enterica* and *E. coli* O157:H7 colonize the surface and internal tissues of plant roots even at very low inoculum doses. A gene expression analysis of roots inoculated with very low doses of *S. enterica* and *E. coli* O157:H7 strains revealed the differential expression of more than 200 probe sets with at least one third of these probe sets commonly up-regulated and one fourth commonly down-regulated in response to both enteric pathogens in plants. Taken together, these results indicate that plants recognize and respond to these enteric pathogens even at very low initial levels of inoculum and these responses were somewhat similar (25–30% similarity) in response to both of these enteric pathogens.

## Materials and Methods

### Bacterial Serovars and Inoculum Preparation

All strains used in this study contain a stably-maintained plasmid with a visible reporter (GFP or *Ds*RED) that is expressed both in culture and *in planta* (Table S1). Single colonies of the strains were isolated and cultured at 37°C for 16 hours in Luria-Bertani (LB) medium supplemented with kanamycin (50 ng/µl) for plasmid maintenance. Overnight cultures were centrifuged and resuspended in phosphate-buffered saline (PBS, pH 7.2). Because it is a common practice to use “cocktails” of serovars when studying human enteric pathogens in plant interactions [40], [41], we prepared two different “cocktails” (one with 5 *S. enterica* serovars and one with 5 *E. coli* O157:H7 serovars; see Table S1) for inoculation, ranging from 2×10^9^ to 2×10^0^ CFU/plant.

### Plasmid Construction and Stability

The plasmid pKT-kan is a broad host-range, stable vector in which a 131-base pair *nptII* promoter fragment from Tn*5* is fused to the *gfp* gene of pPROBE-KT[42]. The *S. enterica* and *E. coli* O157:H7 serovars (Table S1) were transformed by electroporation with pKT-kan.

### Seed Sterilization, Germination, and Inoculation

Medicago seeds were acid-scarified and surface-sterilized, plated on 1% agar supplemented with 1 µg/ml gibberellic acid (GA3), vernalized at 4°C for three days, and allowed to germinate overnight at room temperature. The germinated seedlings were placed in growth pouches (10 seedlings/pouch) containing 15 ml of nitrogen-free Fåhraeus medium as previously described [43]. The seedlings were allowed to grow for one day and were inoculated with 2 ml of cocktail (doses from 2×10^9^ to 2×10^0^ CFU/plant). The plants were grown under 24-hour light conditions at 25°C and were harvested 10 days post-inoculation.

### Determination of the Microbial Population Inside the Root

The protocol for surface sterilization was adapted from Dong *et al.* (2003). Briefly, ten days post inoculation, the seedlings were carefully removed from the growth pouches and immersed in 25 ml of surface sterilization solution (1× PBS, 1% bleach, 0.1% sodium dodecyl sulfate, 0.2% Tween 20). The seedlings in the surface sterilization solution were vortexed vigorously for 1 min followed by 5 washes with 25 ml of sterile water. The efficacy of the surface sterilization was determined by placing the sterilized roots on LB agar plates supplemented with kanamycin (50 ng/µl), followed by incubation at 37°C for 25 min. The roots were removed and the plates were incubated overnight at 37°C for 24 hours as previously described [44]. The roots were crushed manually using a mortar and pestle for 1 min, following which the homogenates were resuspended in 1 ml of 1× PBS containing 20% glycerol, serially diluted in 10-fold increments, and plated on LB agar plates supplemented with kanamycin (50 ng/µl). The plates were incubated at 37°C for 24 hours to enumerate the microbial populations inside the surface sterilized roots. A minimum of 5 plants for each dilution were used to determine the microbial population in the internal tissues.

### Fixation of Roots and Confocal Microscopy

At least 5 seedlings per dilution were used for the fixation and subsequent observation under a ZeissSM 510 Meta scanning confocal laser microscope (SCLM). The roots were fixed in 4% paraformaldehyde solution for 15 min and washed three times with PIPES buffer (pH 7.2). The efficiency of fixation was determined by growing the fixed roots in liquid LB medium supplemented with kanamycin (50 ng/µl) for two days at 37°C. The fluorophores GFP and *Ds*RED were excited at 488 nm and 556 nm, respectively, in the SCLM. The GFP signal was received at 500–510 nm and that of *Ds*RED was received at 583 nm. The fluorescence and bright field images that were obtained were merged together to demarcate the cell boundaries.

### Determination of Bacterial Growth in Fåhraeus Medium and Root Exudates

A single colony of each of the bacterial serotypes was isolated to begin an overnight culture at 37°C. The *S. enterica* and *E. coli* O157:H7 cocktails were prepared by mixing equal proportions of their respective cultures. The growth of these cocktails was monitored spectrophotometrically at 600 nm using a Bioscreen analyzer at 25°C for 72 hours (Labsystems, Helsinki, Finland). As a control, growth in Luria-Bertani (LB) medium was monitored. To test the growth of these enteric pathogens in Medicago root exudates, acid-scarified and surface-sterilized seeds were grown in growth pouches with Fåhraeus medium for 2 weeks at 25°C. The pouches were watered with 5 ml of water once a week, and at the end of 2 weeks, the root exudates were collected and used for determining bacterial growth as mentioned before.

### Gene Expression Analysis

For gene expression analysis, each pouch (10 seedlings/pouch) was inoculated with 2×10^0^ CFU/plant of either the *S. enterica* or *E. coli* O157:H7 cocktail. Plants that were mock-inoculated with 1× PBS were used as controls. RNA was extracted from 4 plants (each from a different pouch) for the control (mock-inoculated) and *S. enterica* and *E. coli* O157:H7 cocktail-inoculated roots using the Qiagen® RNeasy plant mini kit (Chatsworth, CA, U.S.A.). The extracted RNA was quantified using a Thermo Scientific Nano Drop 1000 Spectrophotometer (Wilmington, DE, U.S.A), and 10 µg RNA was used for microarray analysis (4 biological replicates). The Affymetrix GeneChip® Medicago Genome Array (Affymetrix, Santa Clara, CA) was used for the expression analysis. The hybridization of the targets to the arrays, the washing of probe arrays, the staining and the scanning were performed according to the manufacturer’s instructions (Affymetrix, Santa Clara, CA). The data were analyzed using the Empirical Bayes (EB) analysis method using R statistical analysis software to identify differentially-expressed genes in *S. enterica* and *E. coli* O157:H7 cocktail-inoculated roots [45]. The hierarchical log-normal expression model was used to calculate the posterior probability for each pattern at a 5% conditional false discovery rate to determine the appropriate threshold (cFD(_T_)). The critical threshold for the *S. enterica* and *E. coli* O157:H7 cocktail-inoculated roots were 0.94. AgriGO, a web-based platform, was used to perform the enrichment analysis of the differentially expressed transcripts [46].

### Quantitative RT-PCR Analysis

Primers were designed in the intron–exon junction, to avoid genomic DNA contamination, and two internal controls, *ACTIN* and *EF1α*, were used (Table S3). Quantitative RT-PCR reactions were performed in triplicate (3 biological and 3 technical replicates) on a MyIQ PCR machine (Bio-Rad, Hercules, CA, U.S.A.) using the SYBR Advantage qPCR mix (Clontech, Palo Alto, CA, U.S.A.). Cycling conditions were 40 cycles at 95°C for 30 seconds and 59°C for 30 seconds. Data were analyzed using the GENEX program (Bio-Rad, Hercules, CA, U.S.A.). The specificity of the PCR amplification procedures was determined by melt curve (from 55°C to 95°C) analysis and by agarose gel electrophoresis. Expression levels of candidate genes were normalized using the average expression of two reference genes, *MtACTIN* and *MtEF1α* [47].

## Results

### Surface Colonization of Medicago Roots by the Enteric Bacteria *S. enterica* and *E. coli* O157:H7

We selected CYG (Crecimiento y Germinación) seed germination pouches with liquid Fåhraeus medium for our study [17], [24], [43]. This method offers many practical advantages: a large number of plants can be handled simultaneously in a small space, the roots can be separated from the medium with no damage, and any contaminated material can be safely and easily disposed of (Fig. S1).

**Figure 1:**
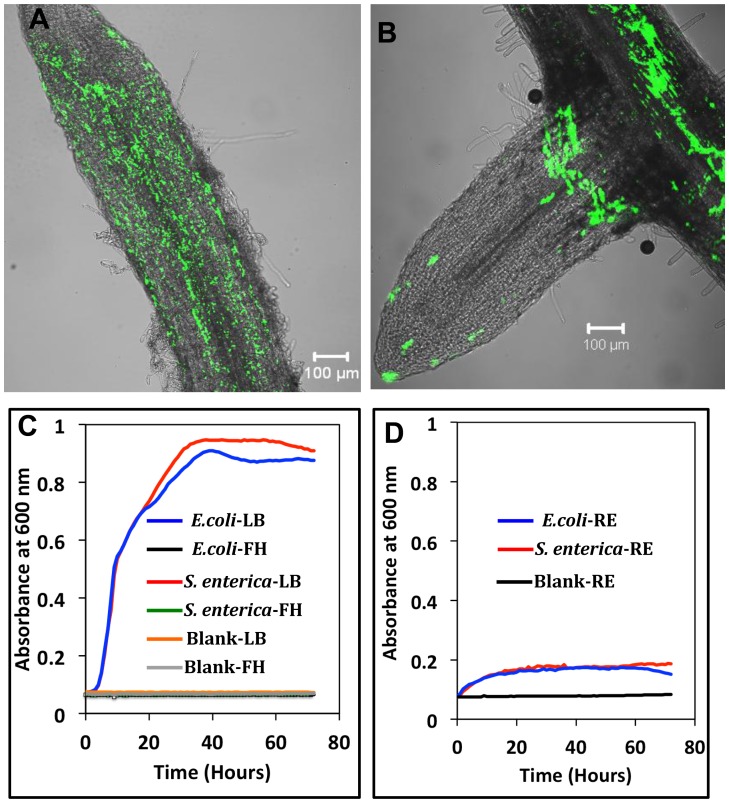
Surface colonization of Medicago roots by cocktails of the enteric pathogens *S. enterica* and *E. coli* O157:H7 and their growth in root exudates. Confocal laser-scanning microscopic image of Medicago roots that were colonized with cocktails of *S. enterica* (A) or *E. coli* O157:H7 (B) containing GFP as the reporter. The plants were inoculated with 1×10^4 ^CFU/plant, and 10 days post-inoculation the roots were fixed with 4% paraformaldehyde and observed under the microscope. Scale bars represent 100 µm. (C) The growth of *S. enterica* and *E. coli* O157:H7 cocktails in Luria-Bertani (LB) and Fåhraeus medium at 25°C for 72 hours. The normal growth of these strains was observed in LB medium, whereas no growth was detected in Fåhraeus medium. (D) The growth of cocktails of *S. enterica* and *E. coli* O157:H7 in 2-week-old root exudates (RE) of Medicago. Both of these cocktails grew in the 2-week-old root exudates.

Germinated Medicago seeds grown in germination pouches were inoculated with cocktails (1×10^4 ^CFU/plant) of either *S. enterica* or *E. coli* O157:H7. All of the strains constitutively expressed either GFP or *Ds*RED [6]. The inoculated roots were fixed for safe handling. An analysis using SCLM confirmed the colonization of Medicago root surfaces preferentially at the sites of lateral root emergence and root tips (Fig. 1A and 1B). These sites of lateral root emergence and the root tips are likely rich in nutrients due to the abundance of root exudates; this richness explains their colonization by enteric bacteria [48]. In order to test the importance of root exudates in supporting the growth of *S. enterica* and *E. coli* O157:H7, the growth of these bacteria in Fåhraeus medium with or without Medicago root exudates was examined. *S. enterica* and *E. coli* O157:H7 showed substantial growth in root exudates, but no growth was observed in Fahräeus medium alone (Fig. 1C and 1D). Collectively, these results suggest that Medicago root exudates may play a role in supporting the growth of these enteric pathogens on the root surface [49].

### Internal Colonization of Medicago Roots by *S. enterica* and *E. coli* O157:H7

**Figure 2:**
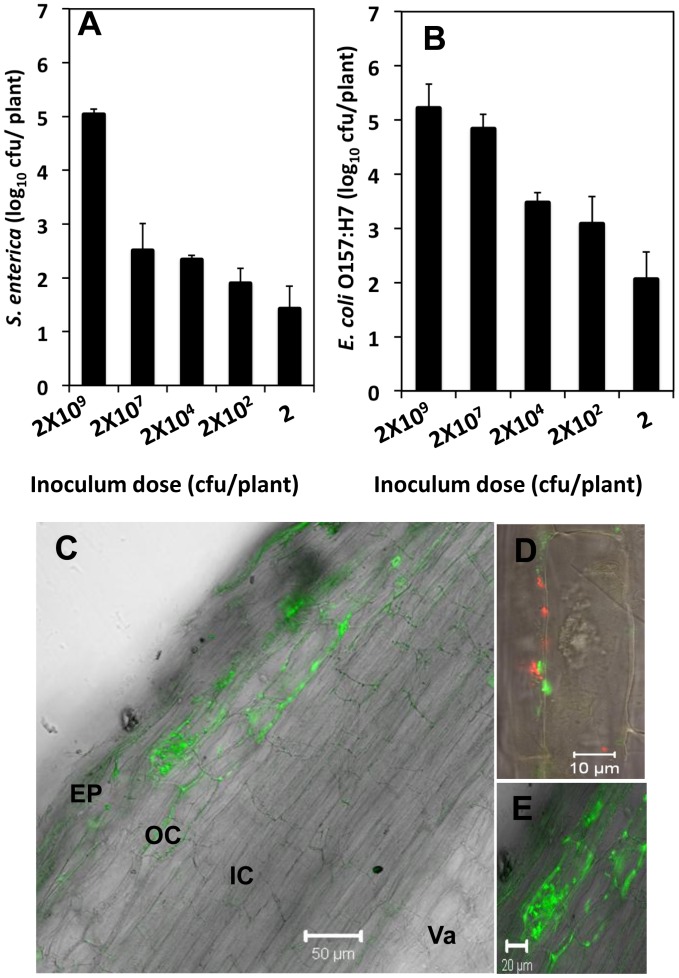
Endophytic colonization of Medicago roots by enteric pathogens (*S. enterica* and *E. coli* O157:H7). Bacterial CFUs recovered from Medicago roots 10 days post-inoculation with cocktails of *S. enterica* (A) and *E. coli* O157:H7 (B). The mean value of 5 replicates is represented; error bars represent standard error of the mean. The CFU values were log_10_ transformed. (Note: 2 ml of inoculum was used.) (C–E) Longitudinal sections of Medicago roots inoculated with enteric pathogens at 1×10^4 ^cfu/plant. At 10 days post-inoculation, the roots were fixed with 4% paraformaldehyde, and sections were obtained using a Vibratome®. (C and E) Longitudinal sections of roots inoculated with the *S. enterica* cocktail. (C) Ep-Epidermis, OC-outer cortex, IC- inner cortex and Va-Vasculature. (D) Longitudinal section of roots inoculated with the *E. coli* O157:H7 cocktail. Note that both strains (*E. coli* O157:H7 expressing either *Ds*RED or GFP as the visible marker) colonize the same region. Scale bar represents 50 µm for C and 10 µm for D and 20 µm for E.

Internal colonization of different plants by *S. enterica* and *E. coli* O157:H7 has been previously reported [17], [50]. However, discrepancies exist in the literature, even when similar experimental conditions were used [11]. Several factors, including plant type and age, bacterial strain and/or serovar, and mode of contamination, may contribute to this variability and influence the internalization of human enteric pathogens in plant tissues [11]. To test whether internalization occurred in our experimental system, surface sterilization was conducted, followed by plate counting and SCLM observations. Surface sterilization and subsequent plate counts were used to quantify the number of bacteria inside of the plant tissues. The plants were inoculated with various doses of inoculum: 2×10^9^, 2×10^6^, 2×10^4^, 2×10^2^ and 2×10^0^ CFU/plant (Fig. 2A and 2B). Ten days post-inoculation, the roots of individual plants were used for plate counts. Both cocktails of enteric bacteria were found to colonize the internal tissues of Medicago roots, even when very low initial inoculum doses were used. An initial inoculum dose of 2×10^0^ CFU/plant was sufficient for colonization in the absence of any competing bacteria, suggesting that the internal contamination of crops can occur under the natural conditions of the food production system where the dose of enteric pathogens may be low. As expected, the higher the initial inoculum dose, the higher the rate of internalization. The internal colonization of Medicago by *E. coli* O157:H7 was consistently higher than that of *S. enterica* (Figure 2A and 2B). However, the exponential relationship between the inoculum dose and the internal colonization rates suggests that both of the enteric pathogens are exposed to unfavorable conditions within the plant tissues and possibly encounter defense reactions [51]–[53]. Finally, at low inoculum doses, variability in the rate of colonization was observed between plants, even though all of the plants had the same genotype and were grown and inoculated under the exact same conditions. This variability indicates that unidentified environmental or physiological factors, along with the probability of contact with the bacteria, affect the colonization process at very low inoculum levels.

In order to confirm internalization and determine the sites of internal colonization at the tissue level, inoculated, surface sterilized and fixed roots were sectioned. Both enteric pathogens were found between epidermal cells, under the epidermal layer and throughout the outer cortex (Fig. 2C), mostly localized in the intercellular spaces (Fig. 2C and 2D). Interestingly, in multiple instances, some outer cortex cells were heavily colonized internally by *S. enterica* (Fig. 2E). These results indicate that both of the enteric pathogens *S. enterica* and *E. coli* O157:H7 are able to enter through the epidermal layer of Medicago roots and colonize the outer cortex very efficiently [54], [55].

### Plant Genes Differentially Regulated during Infection

In most studies of human enteric pathogen association with plants, the focus was primarily on the microbial partner, with less attention given to the plant responses [18], [56]. While more recent studies have focused on the plant responses, some of them unfortunately used relatively high inoculum doses coupled with methods of inoculation that are likely to cause mechanical damage [51], [55]. However from these recent studies we have determined that *S. enterica* and *E. coli* O157:H7 elicit different responses depending on the plant genotype, thereby supporting the hypothesis that the host plant plays a role in regulating these interactions [57], [58].

**Figure 3:**
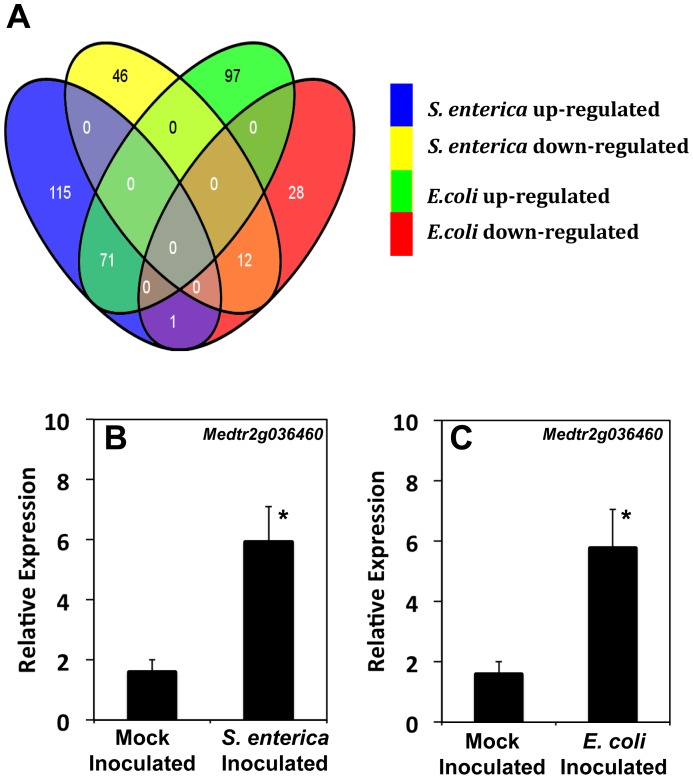
Medicago Affymetrix GeneChip® probe sets differentially expressed in response to inoculation with cocktails of *S. enterica* and *E. coli* O157:H7 and validation of microarray analysis by quantitative RT-PCR. An expression analysis was performed using the Medicago Affymetrix GeneChip® Medicago genome array on 10-day post-inoculated roots inoculated with 2×10^0^ CFU/plant (A). The analysis of the microarray data using EB array statistics identified 187 and 168 probe sets that were up-regulated log 2-fold and 58 and 41 probe sets that were down-regulated when treated with *S. enterica* and *E. coli* O157, respectively. A total of 71 and 12 probe sets were commonly up-and down-regulated, respectively, in both treatments. An expression analysis of 10-day post-inoculated Medicago plants inoculated with 2×10^0^ CFU/plant of a cocktail of either *S. enterica* (B) or *E. coli* O157:H7 (C). The relative expression of kinase Medtr2g036460.1 in the plants inoculated with *S. enterica* (B) and *E. coli* O157:H7 (C). Error bars represent the standard error of the mean from three biological replicates, and * indicates the significance at the 5% level with a *p* value of 0.03 and 0.04 for *S. enterica* and *E. coli* O157:H7 infection, respectively.

In order to identify the plant genes that are regulated in response to relevant inocula of enteric bacteria, expression analyses were performed using GeneChip® Medicago genome Arrays (Affymetrix, Santa Clara, CA). In order to evaluate biologically-relevant conditions, the plants were inoculated with 2×10^0^ CFU/plant of *S. enterica* or *E. coli* O157:H7 cocktails and grown for 10 days in germination pouches. A total of 209 and 245 probe sets were differentially expressed (log 2-fold and above) in *E. coli* O157:H7- and *S. enterica*-inoculated plants, respectively. Among these differentially-expressed probe sets, 71 were commonly up-regulated and 12 were commonly down-regulated by both *E. coli* O157:H7 and *S. enterica.* The common regulation of an overlapping set of differentially expressed genes in Medicago indicates that the mechanism of plant invasion in part may be similar in these two human pathogens (Fig. 3A). A quantitative RT-PCR analysis was performed on individual plants to validate the microarray results. The expression levels for a putative kinase (Medtr2g036460, Fig. 3B and 3C) and two putative lipoxygenases (Medtr8g021750, Fig. S2A and S2B; Medtr8g021690, Fig. S2C and S2D) were monitored in plants inoculated with *S. enterica* or *E. coli* O157:H7. All of the probe sets showed differential expression (up-regulation) in the inoculated roots when compared to the un-inoculated control roots, thereby confirming the microarray data. Here again, variability within the same replicate between plants of the same genotype and which were inoculated under the same conditions was observed. This variability may be due to the use of a low inoculation dose and correlates with our plate count observations.

**Figure 4:**
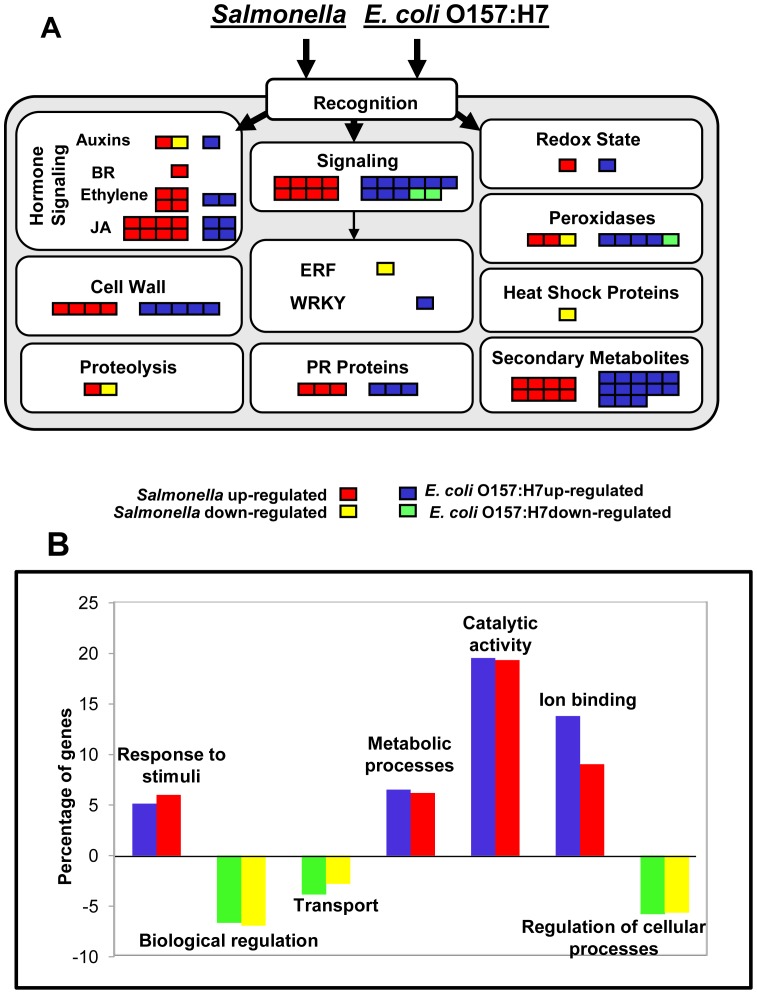
Overview of differentially-expressed probe sets involved in the biotic stress response to inoculation with enteric pathogens and Gene Ontology analysis of over- and under-represented functional groups in the differentially-expressed probe sets. Most of the differentially-expressed probe sets involved in response to inoculation with cocktails of *S. enterica* and *E. coli* O157:H7 play a role in signaling, secondary metabolites, hormone signaling, cell wall modifications, and pathogenesis-related (PR) proteins (A). Gene enrichment analysis using the AgriGO software platform indicates the functional categories over- and under-represented against the reference (B). (Note: the data for the enrichment analysis were normalized against the reference).

Strikingly, many genes that were identified in this microarray experiment seem to play a role in plant basal defenses. The differentially-expressed probe sets were classified using the MapMan platform to obtain an overview of the predicted gene functions related to the pathogen or biotic stress responses in Medicago. More than half of the genes that were related to pathogen defense were regulated similarly in response to *E. coli* O157:H7 and *S. enterica* (Fig. 4A). A total of 17 out of 83 probe sets showed common differential expression in *E. coli* O157:H7- and *S. enterica*-inoculated roots for genes that were predicted to be involved in biotic stress responses (Table 1 and Table 2). Other probe sets that were related to biotic stress responses and that showed a significant differential expression but that were different between these enteric pathogens are listed in Table S2A and S2B.

**Table 1:** List of the probe sets related to the biotic stress response that were differentially expressed in both *E. coli* O157:H7- and *S. enterica*-inoculated plants.

Probe set ID	IMGAG Annotation	Description	Log_2_ FC(*E. coli*O157:H7)	Log_2_ FC(*S. enterica*)
**Secondary metabolism**				
mtr. 20438.1.s1_at	Medtr4g047310.1Medtr4g047310.2Medtr4g047310.3	O-methyltransferase 1 (OMT1/ATOMT1)(Phenylpropanoids/lignin biosynthesis)	2.4	3.2
mtr.14428.1.s1_x_at	Medtr3g105280.1	Chalcone synthase (CHS/TT4/ATCHS)(flavonoids-chalcones)	2.4	2.3
mtr.14428.1.s1_at	Medtr3g105280.1	Chalcone synthase (CHS/TT4/ATCHS)(flavonoids-chalcones)	2.3	2.2
mtr.20567.1.s1_at	Medtr3g105290.1	Chalcone synthase (CHS/TT4/ATCHS)(flavonoids-chalcones)	2.9	2.4
mtr.42370.1.s1_s_at	Medtr8g105350.1Medtr8g105350.2Medtr8g105360.2	Oxidoreductase 2OG-Fe(II) oxygenasefamily protein (flavonoids-flavonols)	2.3	2.1
**PR proteins**				
mtr.34717.1.s1_at	Medtr3g040700.1	Disease resistance-responsive family protein	2.5	2.4
**Peroxidase**				
mtr.40121.1.s1_at	Ac234842_16.1	Peroxidase 22 (PER22/P22/PRXEA)/basic peroxidase E	3.1	2.2
mtr.42373.1.s1_at	Medtr8g136930.1	Peroxidase 40 (PER40/P40)	−2.3	−2.5
mtr.18570.1.s1_at	Medtr5g014310.1	Peroxidase 20 (PER20/P20)	2.1	2.1
**Signaling**				
mtr.9308.1.s1_at	Medtr1g127100.1	Putative photoassimilate-responsive protein(sugar and nutrient physiology)	2.8	2.0
mtr.10454.1.s1_at	Medtr7g111400.1	Polygalacturonase inhibiting protein 1 (PGIP1)(leucine-rich repeat [LRR] XI receptor kinase)	2.1	2.8

The functional categories are in bold. IMGAG Annotation: Gene model predicted by International Medicago Genome Annotation Group (IMGAG) associated with the Probe set ID. Log_2_ FC: Fold change expressed in Log_2_.

**Table 2:** List of the probe sets related to the biotic stress response that were differentially expressed in both *E. coli* O157:H7- and *S. enterica*-inoculated plants.

Probe set ID	IMGAG Annotation	Description	Log_2_ FC (*E. coli*O157:H7)	Log_2_ FC(*S. enterica*)
**Jasmonic Acid**				
mtr.50430.1.s1_at	Medtr8g021690.1	LOX5 lipoxygenase(jasmonate synthesis - degradation)	4.6	6.3
mtr.5628.1.s1_s_at	Medtr8g021750.1 Medtr8g021750.2	LOX1 lipoxygenase(jasmonate synthesis - degradation)	3.4	3.9
mtr.46863.1.s1_s_at	Medtr8g021550.1	LOX5 lipoxygenase(jasmonate synthesis - degradation)	2.1	2.4
mtr.50426.1.s1_at	Medtr8g021750.1 Medtr8g021750.2	LOX1 lipoxygenase(jasmonate synthesis - degradation)	3.9	3.9
**Ethylene**				
mtr.46283.1.s1_s_at	Medtr2g088460.1	Putative 2-oxoglutarate-dependent dioxygenase(ethylene synthesis - degradation)	2.7	2.2
**Auxin**				
mtr.12349.1.s1_at	Medtr7g140870.1	Oxidoreductase (ATB2) (induced-regulated-responsive-activated)	2.2	2.7

The functional categories are in bold. IMGAG Annotation: Gene model predicted by International Medicago Genome Annotation Group (IMGAG) associated with the Probe set ID. Log_2_ FC: Fold change expressed in Log_2._

A total of 185 out of 245 and 158 out of 209 differentially-expressed probe sets of *S. enterica-* and *E. coli* O157:H7-treated plants, respectively, were recognized by Gene Ontology (GO) analysis in the AgriGO platform, which contains 31,225 GO terms for Medicago. Among these recognized GO terms, 42 and 46 showed significant over- or under-representation at the false discovery rate of <5% for *S. enterica* and *E. coli* O157:H7 treatments, respectively. The “response to stimuli” (GO: 0050896), “metabolic processes” (GO: 0008152), “catalytic activity” (GO: 0003824) and “regulation of cellular processes” (GO: 0050794) functional categories were significantly enriched among the differentially-expressed transcripts in both treatments, while the functional categories including “biological regulation” (GO: 0065007), “transport” (GO:0006810) and “regulation of cellular processes” (GO: 0050794) were significantly under-represented compared to the reference for both treatments (Fig. 4B).

Altogether, these results indicate that plants recognize *S. enterica* and *E. coli* O157:H7 with some degree of similarity, in even when inoculated at very low doses (2×10^0^ CFU/plant) and in the absence of mechanical damage.

## Discussion

In recent decades, several studies have been performed to understand the mechanisms that govern plant colonization by human bacterial pathogens [59]. Most of these studies were performed with high doses of human bacterial pathogens [17]. Additionally, in some of the previous reports, the plants were inoculated by mechanical techniques (e.g. infiltration) and in the presence of detergents (e.g. Silwet L-77 surfactant) that facilitate bacterial entry into plant tissues. Different reports indicate that seeds are the most likely contamination of seedlings [60]. Although sprouting seeds represent very low-moisture foods, contaminated seeds still represent a safety concern, as it is well known that *S. enterica* can persist for extended periods of time in low-moisture foods. Under these conditions, a few pathogenic cells present on the seeds can multiply to potentially hazardous levels due to the favorable conditions of moisture, temperature and available nutrients during the subsequent germination and sprouting processes [61]. Additionally, seed decontamination by chemical treatments (e.g. hypochlorite and hydrogen peroxide) can only reduce pathogen levels on seeds but cannot ensure complete elimination because pathogens colonize internal tissues which are not reached by chemical treatments. This is relevant considering that even relatively low levels of surviving pathogens (<0.1 CFU/g) can grow to densities in excess of 1×10^6 ^CFU/g only 48 hours into the sprouting process [37]. Therefore, we performed our experiments with very low inoculum levels (2×10^0^ CFU/plant).

Several studies have reported endophytic colonization by enteric bacteria, but the environmental and physiological factors affecting bacterial entry are still unclear [62]–[64]. Plants harbor a variety of microbes which may positively or negatively impact colonization by human enteric pathogens [65]. Higher populations of competing microbes in the community reduce the rate of colonization by enteric pathogens and vice versa. The attachment of enteric bacteria to plant surfaces seems to be a prerequisite for internal colonization [59]. Both *S. enterica* and *E. coli* O157:H7 attach to plant surfaces using cellulose and aggregative fimbriae [66]–[68]. Attachment using cellulose fibrils by enteric bacteria is similar to that of plant symbiotic and phytopathogenic bacteria [69]. In the present work, the preferential colonization of enteric bacteria was found primarily in three regions (Figure 1A and 1B): (i) at the point of emergence of lateral roots, (ii) near the root cap and (iii) in the intercellular spaces. The roots secrete high amounts of exudates at both the point of the emergence of lateral roots and the root cap, thereby facilitating the survival of a wide range of microbes around this region [70]. Furthermore, the mucilage in the intercellular regions may aid in the proliferation of bacterial cells, resulting in the preferential colonization of enteric bacteria [70].

Bacteria may enter the roots through cracks at the sites of lateral root emergence. In the present study, plate count methods confirmed the internal colonization of Medicago roots by *S. enterica* and *E. coli* O157:H7 (Figure 2A and 2B). At low inoculum doses, significant variability in the rates of colonization was observed for both of these human enteric pathogens, even though the same plant genotype and identical growth and inoculation conditions were used for the study. One possible explanation for this variability is that the rate of colonization may be affected by factors such as the inherent vigor and resistance of the individual seed/seedling and the amount of lateral root formation per plant. These pathogens were able to enter through the epidermis and colonize the outer cortex region of the roots. These enteric pathogens might have used a damaged cell or have actively passed through the epidermis and colonized the outer cortex [55]. Interestingly, no colonization was observed in the inner cortical region, suggesting that the bacteria were not able to reach the vasculature and spread systemically. This lack of colonization may be due to the short time period (10 days) between the inoculation and sectioning. However, we wanted to use a time frame which mirrors the contamination of commercial legume seedlings. The majority of bacterial cells in the colonized outer cortex were found in the apoplastic space (Figure 2C, 2D and 2E). Here, these enteric pathogens may exploit the plant nutritionally by utilizing the polysaccharide components of the cell wall. Interestingly, certain cells in the outer cortex were highly colonized intracellularly by *S. enterica*. A similar colonization pattern was observed by Schikora *et al.* (2008), when studying *Salmonella enterica* serovar Typhimurium in the root hairs of *Arabidopsis thaliana*, by Dong *et al.* (2003), when studying different *S. enterica* serovars in alfalfa and Medicago roots, and by Kutter S, Hartmann A and Schmid M (2006), in barley roots inoculated by *S. enterica* and *Listeria*. Although it was not possible to assess the viability of these cells, it is possible that *Salmonella* may occasionally behave as a saprophyte or even a necrotrophic pathogen.

In order to identify the host genetic factors governing the internal colonization of plant tissues by enteric bacteria, a gene expression analysis was performed. A low inoculum dose was used (2×10^0^ CFU/plant) in order to mimic a biologically relevant exposure in a food production system. Although we used a very low initial inoculum dose of 2×10^0^ CFU/plant, it is likely that the bacterial population has grown in the root exudates (Figure 1Cand 1D) due to the time lag between the inoculation and sampling for microarray analyses. We found similar categories of genes (in terms of cell function) that are differentially expressed in response to inoculation with cocktails of both species of enteric pathogens, indicating that the infection of Medicago plants by *S. enterica* and *E. coli* O157:H7 may in part involve similar mechanisms (Fig. 4A). Approximately one third of the probe sets that are up-regulated in response to *S. enterica* are also up-regulated in response to *E. coli* O157:H7, and approximately one fourth of the probe sets that are down-regulated in response to *S. enterica* are also down-regulated in response to *E. coli* O157:H7. The similarity of responses to the “cocktails” of both of these enteric pathogens suggests that there were no serovar-dependent responses. As such, the use of 2×00 CFU/plant from the five serotype cocktail may not significantly impact the plant response. A gene enrichment analysis of both treatments yielded similar patterns, further suggesting that both of these enteric pathogens may follow similar modes of infection (Fig. 4B). Most of the genes that were differentially expressed in our study are associated with defense reactions, including pathogenesis-related (PR) proteins, peroxidases, cell wall modification components, protein kinases, and plant hormones. Because these transcriptional changes occur 10 days post-inoculation, they may very well represent the adaptive responses of the plants rather than basal defense mechanism.

PR proteins are important components of the plant defense mechanisms that are activated upon pathogen attack [71]. At least three different PR proteins were up-regulated upon infection by *E. coli* O157:H7 and *S. enterica* (Table 1, Table 2, Table S2A and S2B). Peroxidases are also differentially-expressed in response to *E. coli* O157:H7 and *S. enterica*. Peroxidases 22 and 20 were up-regulated, whereas peroxidase 40 was down-regulated by both pathogens (Table 1 and Table 2). At least three more putative peroxidases were up-regulated in response to *E. coli* O157:H7 infection (Table S2B). Modification of the cell wall is a common characteristic of the plant defense response [72]. Several genes associated with cell wall modification and secondary metabolisms were differentially expressed, with the majority showing up-regulation during infection (Table 1, Table 2, Table S2A and S2B). In *E. coli* O157:H7-infected roots, pectate lyase and expansin genes were up-regulated. Cellulose synthase-like, fasciclin-like arabinogalactan-protein, and putative pectin esterase are up-regulated in *S. enterica-*infected roots. Pectin esterase in particular is implicated in the modification of plant cell walls by altering the local pH.

Leucine-rich repeat receptor kinases, lectin protein kinase family proteins, and several members of the protein kinase family were up-regulated, while serine/threonine kinases and cysteine–rich receptor-like protein kinases were down-regulated during *E. coli* O157:H7 infection (Table 1, Table 2, and Table S2B). Infection by *S. enterica* led to the up-regulation of a wide array of kinases, including a leucine-rich repeat receptor kinase, a histidine phospho-transfer kinase and a putative wall-associated receptor kinase-like precursor (Table S2A). Among these kinases, wall-associated receptor kinase-like proteins have been implicated in cell elongation, plant development, and plant responses to pathogens [73], [74]. Differentially-expressed leucine-rich repeat receptor kinases may be candidates for MAMP recognition receptors [56].

The plant defense response can be very broadly classified into salicylic acid-dependent (SA) and jasmonic acid (JA)/ethylene-dependent responses. SA-dependent pathways are generally activated in response to biotrophic pathogens whereas, JA-dependent pathways are generally implicated in wound responses and defense against herbivore attacks and necrotrophic pathogens. However, plant defense responses often involve a cross talk between the SA- and JA-dependent pathways that is often modulated by ethylene [75]. In this study, many genes in the JA and ethylene pathways were differentially regulated in response to *S. enterica* and *E. coli* O157:H7 (Fig. 4A and 4B). Lipoxygenases 1 and 5 (LOX1 and LOX5) were induced by both organisms. Another gene involved in the JA pathway, 12-oxophytodienoate reductase 2 (OPR2), was up-regulated in response to *S. enterica* [76]. Zinc finger transcription factors and ethylene-responsive transcription factors (AP2/ERF family) were induced in both inoculations (Table S2A and S2B). Ethylene is an important component of the plant defense response to pathogens. In Medicago, the ethylene-insensitive mutant *sickle* was more heavily colonized by *S. enterica* serovar Typhimurium than were wild-type plants [77]–[79]. Reciprocally, the rate of colonization was significantly lower in plants that were grown on a medium that was supplemented with an ethylene precursor (1-aminocyclopropane-1-carboxylic acid), demonstrating the inhibitory role of ethylene in endophytic colonization [77]. At least 2 genes corresponding to ethylene synthesis and degradation in *E. coli* O157:H7-infected roots and 3 genes in *S. enterica*-infected roots were up-regulated (Table1, Table 2, Table S2A and Table S2B). Interestingly, no probe set corresponding to SA-dependent pathways was regulated during infection by *E. coli* O157:H7 or *S. enterica*. Altogether, these results suggest that the JA and ethylene pathways are the main pathways regulated in Medicago in response to human enteric pathogens. The fact that we observed some cells heavily colonized intracellularly also suggests that *S. enterica* may behave as a saprophyte or as a necrotrophic pathogen.

These results strongly correlate with those from a previous study in *Arabidopsis thaliana* leaves [54]. In our study, the plants were not mechanically damaged by the germination pouch procedure and were inoculated with a low inoculum (2×10^0^ CFU/plant). A major difference with Schikora *et al.* (2008) is that they used *Arabidopsis thaliana* leaves, whereas we used Medicago roots. Leaves are known to be a hostile environment for bacteria, whereas roots, and especially legume roots, produce large amounts of root exudates that attract and feed microbial communities. Legume roots associate with nitrogen-fixing soil bacteria called rhizobia and may be fairly permissive for infection by other bacteria. This may explain why infections related to the consumption of legume seedlings are so frequent [7]. Moreover, the fact that these human pathogens can enter through the epidermal layer and colonize the outer cortex explains why the current surface sterilization procedures cannot efficiently prevent these infections.

In the short term, the identification of plant genes that are regulated upon infection by low inoculum levels of *S. enterica* and *E. coli* O157:H7 may provide useful markers for the rapid and sensitive detection of crop contamination with human pathogens. In the long term, studies of plant responses to these bacteria may lead to the development of crops that are more resistant to colonization by human and plant pathogens at early infection stages. Our results suggest that a broad increase in resistance may also mount defenses against beneficial bacteria such as rhizobia. Although the rhizobium–legume interaction association is dispensable for plant growth, this symbiotic association is highly beneficial for agricultural sustainability [80]. It will, therefore, be important to pursue efforts toward the identification or engineering of mechanisms allowing plants to specifically recognize bacterial human pathogens while maintaining associations with beneficial microbes.

## Supporting Information

Figure S1 **Experimental systems used for studying interaction of human enteric pathogens** ***Salmonella enterica*** **and** ***E. coli*** **O157:H7 with Medicago.** Germinated Jemalong A17 seedlings were planted in growth pouches filled with modified Fahraeus medium (A and B). Seedlings were inoculated with enteric pathogens *Salmonella* and *E. coli* O157:H7 the next day. Individual plant was removed 10 days post-inoculation and tested for surface and internal colonization.
(TIF)

Figure S2 **Validation of Medicago Affymetrix GeneChip® probe sets differentially expressed in response to inoculation with** ***Salmonella enterica*** **and** ***E. coli*** **O157:H7 cocktails by Quantitative RT-PCR.** Expression analysis of 10 days post inoculation Medicago Jemalong A17 plants inoculated with 2 cfu/plant of either *Salmonella* (A and C) or *E. coli* O157:H7 (B and D) cocktail. Relative expression of lipooxygenases Medtr8g021750 and Medtr8g021690 in plants inoculated with *Salmonella* (A and C) and *E. coli* O157:H7 (B and D). Error bar represent the standard error of mean from three biological replicates.
(TIF)

Table S1 **List of bacterial serovars used for this study and the corresponding markers in their plasmid.**
(DOCX)

Table S2 **Probesets involved in biotic stress response with differential expression in** ***Salmonella enterica*** **and** ***E.coli*** **O157:H7 inoculated plants.** The functional categories are in bold. IMGAG Annotation: Gene model predicted by International Medicago Genome Annotation Group (IMGAG) associated with the Probe set ID. Fold Change: Fold change expressed in Log2. *A: Probesets involved in biotic stress response with differential expression in Salmonella enterica inoculated plants.* The functional categories are in bold. IMGAG Annotation: Gene model predicted by International Medicago Genome Annotation Group (IMGAG) associated with the Probe set ID. Fold Change: Fold change expressed in Log2. *B: Probesets involved in biotic stress response with differential expression in E.coli O157:H7 inoculated plants.* The functional categories are in bold. IMGAG Annotation: Gene model predicted by International Medicago Genome Annotation Group (IMGAG) associated with the Probe set ID. Fold Change: Fold change expressed in Log2.
(DOCX)

Table S3 **List of primers used for Quantitative RT-PCR.**
(DOCX)

## References

[1] Berger C, Sodha S, Shaw R, Griffin P, Pink D, et al (2010) Fresh fruit and vegetables as vehicles for the transmission of human pathogens. Environmental Microbiology 12: 2385–2397. 20636374 10.1111/j.1462-2920.2010.02297.x

[2] Pollack S (2001) Consumer demand for fruit and vegetables: the US example: economic research service. Washington, DC, USA.: United States Department of Agriculture.

[3] CDC (2011) Surveillance for foodborne disease outbreaks - United States. 2008. MMWR Morb Mortal Wrkly. 1197–1202. 21900873

[4] Sivapalasingam S, Friedman C, Cohen L, Tauxe R (2004) Fresh produce: a growing cause of outbreaks of foodborne illness in the United States, 1973 through 1997. Journal of Food Protection 67: 2342–2353. 15508656 10.4315/0362-028x-67.10.2342

[5] Batz MB, Morris JG (2011) Ranking the risks: top 10 pathogen-food combinations with the greatest burden on public health.

[6] Barak JD, Whitehand LC, Charkowski AO (2002) Differences in attachment of *Salmonella enterica* serovars and *Escherichia coli* O157:H7 to alfalfa sprouts. Applied and Environmental Microbiology 68: 4758–4763. 12324317 10.1128/AEM.68.10.4758-4763.2002 PMC126431

[7] Mohle-Boetani JC, Farrar J, Bradley P, Barak JD, Miller M, et al (2009) *Salmonella* infections associated with mung bean sprouts: epidemiological and environmental investigations. Epidemiology and Infection 137: 357–366. 18294429 10.1017/S0950268808000411

[8] Wachtel M, Charkowski A (2002) Cross-contamination of lettuce with *Escherichia coli* O157 : H7. Journal of Food Protection 65: 465–470. 11899044 10.4315/0362-028x-65.3.465

[9] Kroupitski Y, Golberg D, Belausov E, Pinto R, Swartzberg D, et al (2009) Internalization of *Salmonella enterica* in leaves is induced by light and involves chemotaxis and penetration through open stomata. Applied and Environmental Microbiology 75: 6076–6086. 19648358 10.1128/AEM.01084-09 PMC2753090

[10] Ukuku D, Sapers G (2007) Effect of time before storage and storage temperature on survival of *Salmonella* inoculated on fresh-cut melons. Food Microbiology 24: 288–295. 17188207 10.1016/j.fm.2006.04.007

[11] Deering AJ, Mauer LJ, Pruitt RE (2012) Internalization of *E. coli* O157:H7 and *Salmonella* spp. in plants: a review. Food Research International 45: 567–575.

[12] Yadav R, Karamanoli K, Vokou D (2005) Bacterial colonization of the phyllosphere of mediterranean perennial species as influenced by leaf structural and chemical features. Microbial Ecology 50: 185–196. 16215646 10.1007/s00248-004-0171-y

[13] Cooley M, Miller W, Mandrell R (2003) Colonization of *Arabidopsis thaliana* with *Salmonella enterica* and enterohemorrhagic *Escherichia coli* O157 : H7 and competition by *Enterobacter asburiae* . Applied and Environmental Microbiology 69: 4915–4926. 12902287 10.1128/AEM.69.8.4915-4926.2003 PMC169118

[14] Lindow S, Brandl M (2003) Microbiology of the phyllosphere. Applied and Environmental Microbiology 69: 1875–1883. 12676659 10.1128/AEM.69.4.1875-1883.2003 PMC154815

[15] Riely B, Mun J, Ane J (2006) Unravelling the molecular basis for symbiotic signal transduction in legumes. Molecular Plant Pathology 7: 197–207. 20507440 10.1111/j.1364-3703.2006.00328.x

[16] Brelles-Mariño G, Ané J (2008) Nod factors and the molecular dialogue in the rhizobia-legume interaction. Nitrogen Fixation Research Progress: Nova Science Publishers, Inc. 173–227.

[17] Dong Y, Iniguez A, Ahmer B, Triplett E (2003) Kinetics and strain specificity of rhizosphere and endophytic colonization by enteric bacteria on seedlings of *Medicago sativa* and *Medicago truncatula* . Applied and Environmental Microbiology 69: 1783–1790. 12620870 10.1128/AEM.69.3.1783-1790.2003 PMC150109

[18] Klerks MM, Franz E, van Gent-Pelzer M, Zijlstra C, van Bruggen AH (2007) Differential interaction of *Salmonella enterica* serovars with lettuce cultivars and plant-microbe factors influencing the colonization efficiency. The ISME journal 1: 620–631. 18043669 10.1038/ismej.2007.82

[19] Rediers H, Bonnecarrere V, Rainey PB, Hamonts K, Vanderleyden J, et al (2003) Development and application of a dapB-based in vivo expression technology system to study colonization of rice by the endophytic nitrogen-fixing bacterium *Pseudomonas stutzeri* A15. Applied and Environmental Microbiology 69: 6864–6874. 14602651 10.1128/AEM.69.11.6864-6874.2003 PMC262291

[20] Pinton R, Varanini Z, Nannipieri P (2001) The rhizosphere: biochemistry and organic substances at the soil-plant interface; Pinton R, Varanini Z, Nannipieri P, editors.

[21] Cook D (1999) *Medicago truncatula* - a model in the making! Commentary. Current Opinion in Plant Biology 2: 301–304. 10459004 10.1016/s1369-5266(99)80053-3

[22] Rose RJ (2008) *Medicago truncatula* as a model for understanding plant interactions with other organisms, plant development and stress biology: past, present and future. Functional Plant Biology 35: 253–264. 10.1071/FP07297 32688781

[23] Thoquet P, Gherardi M, Journet E, Kereszt A, Ane J, et al (2002) The molecular genetic linkage map of the model legume *Medicago truncatula*: an essential tool for comparative legume genomics and the isolation of agronomically important genes. BMC Plant Biology 2: 1. 11825338 10.1186/1471-2229-2-1 PMC65051

[24] Boisson-Dernier A, Chabaud M, Garcia F, Becard G, Rosenberg C, et al (2001) *Agrobacterium rhizogenes*-transformed roots of *Medicago truncatula* for the study of nitrogen-fixing and endomycorrhizal symbiotic associations. Molecular Plant-Microbe Interactions 14: 695–700. 11386364 10.1094/MPMI.2001.14.6.695

[25] Chabaud M, de Carvalho-Niebel F, Barker D (2003) Efficient transformation of *Medicago truncatula* cv. Jemalong using the hypervirulent *Agrobacterium tumefaciens* strain AGL1. Plant Cell Reports 22: 46–51. 12827434 10.1007/s00299-003-0649-y

[26] Tadege M, Ratet P, Mysore K (2005) Insertional mutagenesis: a swiss army knife for functional genomics of *Medicago truncatula* . Trends in Plant Science 10: 229–235. 15882655 10.1016/j.tplants.2005.03.009

[27] Tadege M, Wen J, He J, Tu H, Kwak Y, et al (2008) Large-scale insertional mutagenesis using the *Tnt1* retrotransposon in the model legume *Medicago truncatula* . Plant Journal 54: 335–347. 18208518 10.1111/j.1365-313X.2008.03418.x

[28] Limpens E, Ramos J, Franken C, Raz V, Compaan B, et al (2004) RNA interference in *Agrobacterium rhizogenes*-transformed roots of Arabidopsis and *Medicago truncatula* . Journal of Experimental Botany 55: 983–992. 15073217 10.1093/jxb/erh122

[29] Constantin G, Krath B, MacFarlane S, Nicolaisen M, Johansen I, et al (2004) Virus-induced gene silencing as a tool for functional genomics in a legume species. Plant Journal 40: 622–631. 15500476 10.1111/j.1365-313X.2004.02233.x

[30] Constantin G, Gronlund M, Johansen I, Stougaard J, Lund O (2008) Virus-induced gene silencing (VIGS) as a reverse genetic tool to study development of symbiotic root nodules. Molecular Plant-Microbe Interactions 21: 720–727. 18624636 10.1094/MPMI-21-6-0720

[31] Kulikova O, Gualtieri G, Geurts R, Kim D, Cook D, et al (2001) Integration of the FISH pachytene and genetic maps of *Medicago truncatula* . Plant Journal 27: 49–58. 11489182 10.1046/j.1365-313x.2001.01057.x

[32] Kulikova O, Geurts R, Lamine M, Kim D, Cook D, et al (2004) Satellite repeats in the functional centromere and pericentromeric heterochromatin of *Medicago truncatula* . Chromosoma 113: 276–283. 15480726 10.1007/s00412-004-0315-3

[33] Young ND, Debelle F, Oldroyd GE, Geurts R, Cannon SB, et al (2011) The *Medicago* genome provides insight into the evolution of rhizobial symbioses. Nature 480: 520–524. 22089132 10.1038/nature10625 PMC3272368

[34] Weissinger W, Beuchat L (2000) Comparison of aqueous chemical treatments to eliminate Salmonella on alfalfa seeds. Journal of Food Protection 63: 1475–1482. 11079686 10.4315/0362-028x-63.11.1475

[35] Weissinger W, Chantarapanont W, Beuchat L (2000) Survival and growth of *Salmonella baildon* in shredded lettuce and diced tomatoes, and effectiveness of chlorinated water as a sanitizer. International Journal of Food Microbiology 62: 123–131. 11139012 10.1016/s0168-1605(00)00415-3

[36] Weissinger W, McWatters K, Beuchat L (2001) Evaluation of volatile chemical treatments for lethality to Salmonella on alfalfa seeds and sprouts. Journal of Food Protection 64: 442–450. 11307877 10.4315/0362-028x-64.4.442

[37] Holliday S, Scouten A, Beuchat L (2001) Efficacy of chemical treatments in eliminating *Salmonella* and *Escherichia coli* O157: H7 on scarified and polished alfalfa seeds. Journal of Food Protection 64: 1489–1495. 11601695 10.4315/0362-028x-64.10.1489

[38] Franz E, Visser AA, Van Diepeningen AD, Klerks MM, Termorshuizen AJ, et al (2007) Quantification of contamination of lettuce by GFP-expressing *Escherichia coli* O157:H7 and *Salmonella enterica* serovar Typhimurium. Food Microbiology 24: 106–112. 16943102 10.1016/j.fm.2006.03.002

[39] Tyler HL, Triplett EW (2008) Plants as a habitat for beneficial and/or human pathogenic bacteria. Annual Review of Phytopathology 46: 53–73. 10.1146/annurev.phyto.011708.103102 18680423

[40] Zheng J, Allard S, Reynolds S, Millner P, Arce G, et al (2013) Colonization and internalization of *Salmonella enterica* in tomato plants. Applied and Environmental Microbiology 79: 2494–2502. 23377940 10.1128/AEM.03704-12 PMC3623171

[41] Hao LY, Willis DK, Andrews-Polymenis H, McClelland M, Barak JD (2012) Requirement of siderophore biosynthesis for plant colonization by *Salmonella enterica* . Applied and Environmental Microbiology 78: 4561–4570. 22522683 10.1128/AEM.07867-11 PMC3370490

[42] Miller W, Leveau J, Lindow S (2000) Improved gfp and inaZ broad-host-range promoter-probe vectors. Molecular Plant-Microbe Interactions 13: 1243–1250. 11059491 10.1094/MPMI.2000.13.11.1243

[43] Catoira R, Galera C, de Billy F, Penmetsa R, Journet E, et al (2000) Four genes of *Medicago truncatula* controlling components of a nod factor transduction pathway. The Plant Cell 12: 1647–1665. 11006338 10.1105/tpc.12.9.1647 PMC149076

[44] Gandhi M, Golding S, Yaron S, Matthews K (2001) Use of green fluorescent protein expressing *Salmonella Stanley* to investigate survival, spatial location, and control on alfalfa sprouts. Journal of Food Protection 64: 1891–1898. 11770613 10.4315/0362-028x-64.12.1891

[45] Edwards JW, Page GP, Gadbury G, Heo M, Kayo T, et al (2005) Empirical Bayes estimation of gene-specific effects in micro-array research. Functional & Integrative Genomics 5: 32–39. 15455262 10.1007/s10142-004-0123-0

[46] Du Z, Zhou X, Ling Y, Zhang Z, Su Z (2010) agriGO: a GO analysis toolkit for the agricultural community. Nucleic Acids Research 38: W64–W70. 20435677 10.1093/nar/gkq310 PMC2896167

[47] Messinese E, Mun J, Yeun L, Jayaraman D, Rouge P, et al (2007) A novel nuclear protein interacts with the symbiotic DMI3 calcium- and calmodulin-dependent protein kinase of *Medicago truncatula* . Molecular Plant-Microbe Interactions 20: 912–921. 17722695 10.1094/MPMI-20-8-0912

[48] Bais HP, Weir TL, Perry LG, Gilroy S, Vivanco JM (2006) The role of root exudates in rhizosphere interactions with plants and other organisms. Annual Review of Plant Biology 57: 233–266. 10.1146/annurev.arplant.57.032905.105159 16669762

[49] Hernández-Reyes C, Schikora A (2013) *Salmonella,* a cross-kingdom pathogen infecting humans and plants. FEMS Microbiology Letters 343: 1–7. 23488473 10.1111/1574-6968.12127

[50] Sharma M, Ingram DT, Patel JR, Millner PD, Wang X, et al (2009) A novel approach to investigate the uptake and internalization of *Escherichia coli* O157:H7 in spinach cultivated in soil and hydroponic medium. Journal of Food Protection 72: 1513–1520. 19681280 10.4315/0362-028x-72.7.1513

[51] Shirron N, Yaron S (2011) Active suppression of early immune response in tobacco by the human pathogen *Salmonella Typhimurium* . PLOS ONE 6: e18855. 21541320 10.1371/journal.pone.0018855 PMC3082535

[52] Meng F, Altier C, Martin G (2013) Salmonella colonization activates the plant immune system and benefits from association with plant pathogenic bacteria. Environmental Microbiology 15: 2418–2430. 23517029 10.1111/1462-2920.12113

[53] Roy D, Panchal S, Rosa B, Melotto M (2013) *Escherichia coli* O157:H7 induces stronger plant immunity than *Salmonella enterica* Typhimurium SL1344. Phytopathology 103: 326–332. 23301812 10.1094/PHYTO-09-12-0230-FI PMC3982233

[54] Schikora A, Carreri A, Charpentier E, Hirt H (2008) The dark side of the salad: *Salmonella typhimurium* overcomes the innate immune response of *Arabidopsis thaliana* and shows an endopathogenic lifestyle. PLOS ONE 3: e2279. 18509467 10.1371/journal.pone.0002279 PMC2386236

[55] Schikora A, Virlogeux-Payant I, Bueso E, Garcia AV, Nilau T, et al (2011) Conservation of *Salmonella* infection mechanisms in plants and animals. PLOS ONE 6: e24112. 21915285 10.1371/journal.pone.0024112 PMC3167816

[56] Thilmony R, Underwood W, He SY (2006) Genome-wide transcriptional analysis of the *Arabidopsis thaliana* interaction with the plant pathogen *Pseudomonas syringae* pv. tomato DC3000 and the human pathogen *Escherichia coli* O157:H7. The Plant Journal 46: 34–53. 16553894 10.1111/j.1365-313X.2006.02725.x

[57] Teplitski M, Warriner K, Bartz J, Schneider KR (2011) Untangling metabolic and communication networks: interactions of enterics with phytobacteria and their implications in produce safety. Trends in Microbiology 19: 121–127. 21177108 10.1016/j.tim.2010.11.007

[58] Teplitski M, Noel JT, Alagely A, Danyluk MD (2012) Functional genomics studies shed light on the nutrition and gene expression of non-typhoidal *Salmonella* and enterovirulent *E. coli* in produce. Salmonella in Foods: Evolution, Strategies and Challenges 45: 576–586.

[59] Schikora A, Garcia AV, Hirt H (2012) Plants as alternative hosts for *Salmonella* . Trends in Plant Science 17: 245–249. 22513107 10.1016/j.tplants.2012.03.007

[60] Fett W, Cooke P (2005) A survey of native microbial aggregates on alfalfa, clover and mung bean sprout cotyledons for thickness as determined by confocal scanning laser microscopy. Food Microbiology 22: 253–259.

[61] Neetoo H, Chen H (2011) Individual and combined application of dry heat with high hydrostatic pressure to inactivate Salmonella and *Escherichia coli* O157:H7 on alfalfa seeds. Food Microbiology 28: 119–127. 21056783 10.1016/j.fm.2010.09.004

[62] Barak JD, Kramer LC, Hao LY (2011) Colonization of tomato plants by *Salmonella enterica* is cultivar dependent, and type 1 trichomes are preferred colonization sites. Applied and Environmental Microbiology 77: 498–504. 21075871 10.1128/AEM.01661-10 PMC3020540

[63] Hora R, Warriner K, Shelp BJ, Griffiths MW (2005) Internalization of *Escherichia coli* O157:H7 following biological and mechanical disruption of growing spinach plants. Journal of Food Protection 68: 2506–2509. 16355819 10.4315/0362-028x-68.12.2506

[64] Warriner K, Spaniolas S, Dickinson M, Wright C, Waites WM (2003) Internalization of bioluminescent *Escherichia coli* and *Salmonella Montevideo* in growing bean sprouts. Journal of Applied Microbiology 95: 719–727. 12969285 10.1046/j.1365-2672.2003.02037.x

[65] Cooley M, Chao D, Mandrell R (2006) *Escherichia coli* O157 : H7 survival and growth on lettuce is altered by the presence of epiphytic bacteria. Journal of Food Protection 69: 2329–2335. 17066909 10.4315/0362-028x-69.10.2329

[66] Barak JD, Gorski L, Naraghi-Arani P, Charkowski AO (2005) *Salmonella enterica* virulence genes are required for bacterial attachment to plant tissue. Applied and Environmental Microbiology 71: 5685–5691. 16204476 10.1128/AEM.71.10.5685-5691.2005 PMC1265987

[67] Barak JD, Jahn CE, Gibson DL, Charkowski AO (2007) The role of cellulose and O-antigen capsule in the colonization of plants by *Salmonella enterica* . Molecular Plant-Microbe Interactions : MPMI 20: 1083–1091. 17849711 10.1094/MPMI-20-9-1083

[68] Teplitski M, Barak JD, Schneider KR (2009) Human enteric pathogens in produce: un-answered ecological questions with direct implications for food safety. Current Opinion in Biotechnology 20: 166–171. 19349159 10.1016/j.copbio.2009.03.002

[69] Rodriguez-Navarro DN, Dardanelli MS, Ruiz-Sainz JE (2007) Attachment of bacteria to the roots of higher plants. FEMS Microbiology Letters 272: 127–136. 17521360 10.1111/j.1574-6968.2007.00761.x

[70] Chaintreuil C, Giraud E, Prin Y, Lorquin J, Ba A, et al (2000) Photosynthetic bradyrhizobia are natural endophytes of the African wild rice *Oryza breviligulata* . Applied and Environmental Microbiology 66: 5437–5447. 11097925 10.1128/aem.66.12.5437-5447.2000 PMC92479

[71] Sels J, Mathys J, De Coninck BM, Cammue BP, De Bolle MF (2008) Plant pathogenesis-related (PR) proteins: a focus on PR peptides. Plant Physiology and Biochemistry : PPB/Societe francaise de physiologie vegetale 46: 941–950. 10.1016/j.plaphy.2008.06.011 18674922

[72] Cantu D, Vicente A, Labavitch J, Bennett A, Powell A (2008) Strangers in the matrix: plant cell walls and pathogen susceptibility. Trends in Plant Science 13: 610–617. 18824396 10.1016/j.tplants.2008.09.002

[73] He Z, Cheeseman I, He D, Kohorn B (1999) A cluster of five cell wall-associated receptor kinase genes, Wak1–5, are expressed in specific organs of Arabidopsis. Plant Molecular Biology 39: 1189–1196. 10380805 10.1023/a:1006197318246

[74] Verica JA, Chae L, Tong H, Ingmire P, He ZH (2003) Tissue-specific and developmentally regulated expression of a cluster of tandemly arrayed cell wall-associated kinase-like kinase genes in *Arabidopsis* . Plant Physiology 133: 1732–1746. 14576286 10.1104/pp.103.028530 PMC300728

[75] Ballare CL (2011) Jasmonate-induced defenses: a tale of intelligence, collaborators and rascals. Trends in Plant Science 16: 249–257. 21216178 10.1016/j.tplants.2010.12.001

[76] Turner JG, Ellis C, Devoto A (2002) The jasmonate signal pathway. The Plant Cell 14 Suppl: S153–164 10.1105/tpc.000679 PMC151253 12045275

[77] Iniguez AL, Dong Y, Carter HD, Ahmer BM, Stone JM, et al (2005) Regulation of enteric endophytic bacterial colonization by plant defenses. Molecular Plant-Microbe Interactions : MPMI 18: 169–178. 15720086 10.1094/MPMI-18-0169

[78] Uppalapati SR, Marek SM, Lee HK, Nakashima J, Tang Y, et al (2009) Global gene expression profiling during *Medicago truncatula*-*Phymatotrichopsis omnivora* interaction reveals a role for jasmonic acid, ethylene, and the flavonoid pathway in disease development. Molecular Plant-Microbe Interactions : MPMI 22: 7–17. 19061398 10.1094/MPMI-22-1-0007

[79] Zimmerli L, Stein M, Lipka V, Schulze-Lefert P, Somerville S (2004) Host and non-host pathogens elicit different jasmonate/ethylene responses in Arabidopsis. The Plant Journal : for cell and molecular biology 40: 633–646. 15546348 10.1111/j.1365-313X.2004.02236.x

[80] Vance C (2001) Symbiotic nitrogen fixation and phosphorus acquisition. Plant nutrition in a world of declining renewable resources. Plant Physiology 127: 390–397. 11598215 PMC1540145

