# Supplementary figures and images for: Response of Medicago truncatula Seedlings to Colonization by Salmonella enterica and Escherichia coli O157:H7
Source: PLoS One. 2014 Feb 14;9(2):e87970. doi: 10.1371/journal.pone.0087970 (PMC3925098; doi:10.1371/journal.pone.0087970)

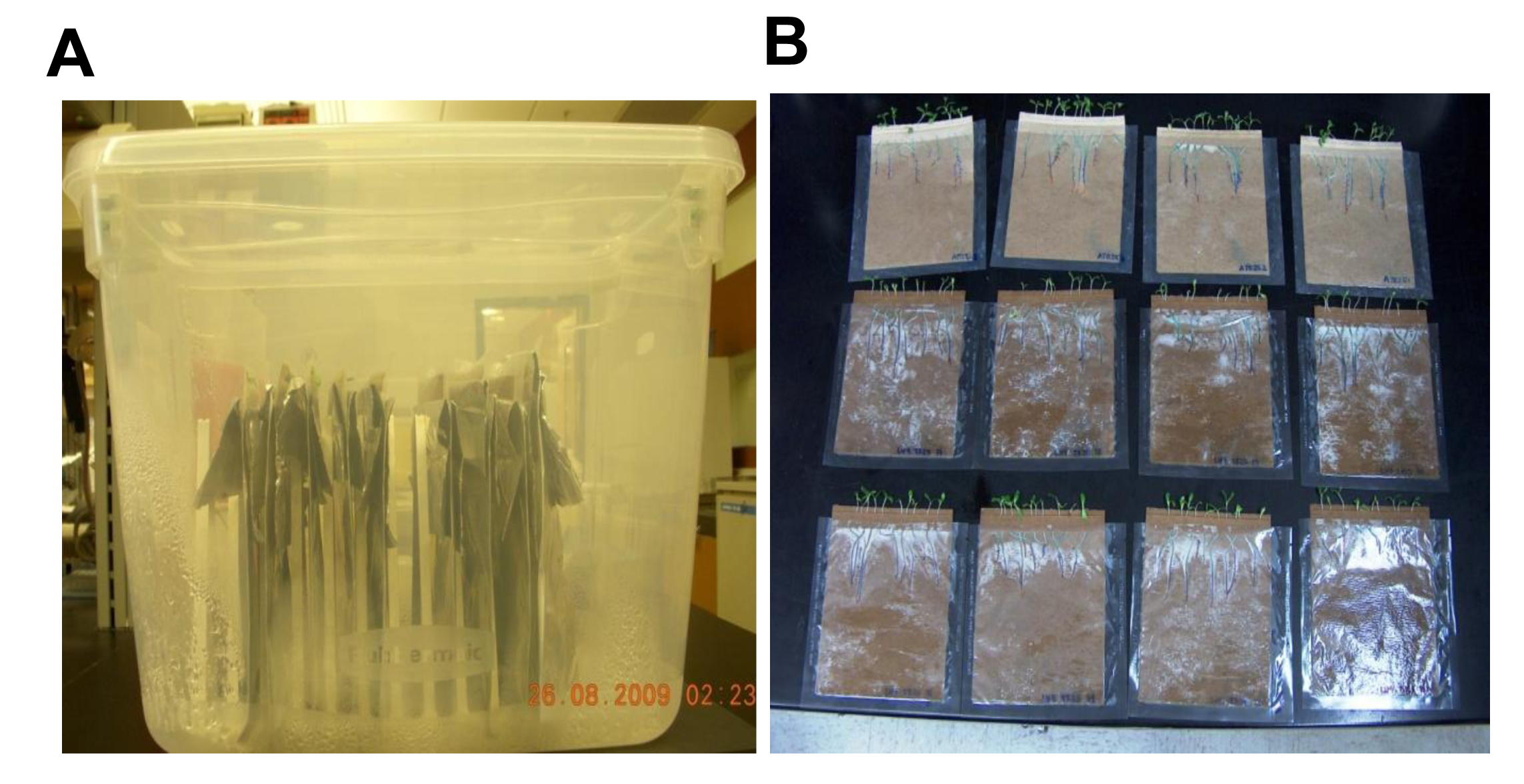

Supplement: Figure S1 — Experimental systems used for studying interaction of human enteric pathogens Salmonella enterica and E. coli O157:H7 with Medicago. Germinated Jemalong A17 seedlings were planted in growth pouches filled with modified Fahraeus medium (A and B). Seedlings were inoculated with enteric pathogens Salmonella and E. coli O157:H7 the next day. Individual plant was removed 10 days post-inoculation and tested for surface and internal colonization. (TIF) [file pone.0087970.s001.tif]

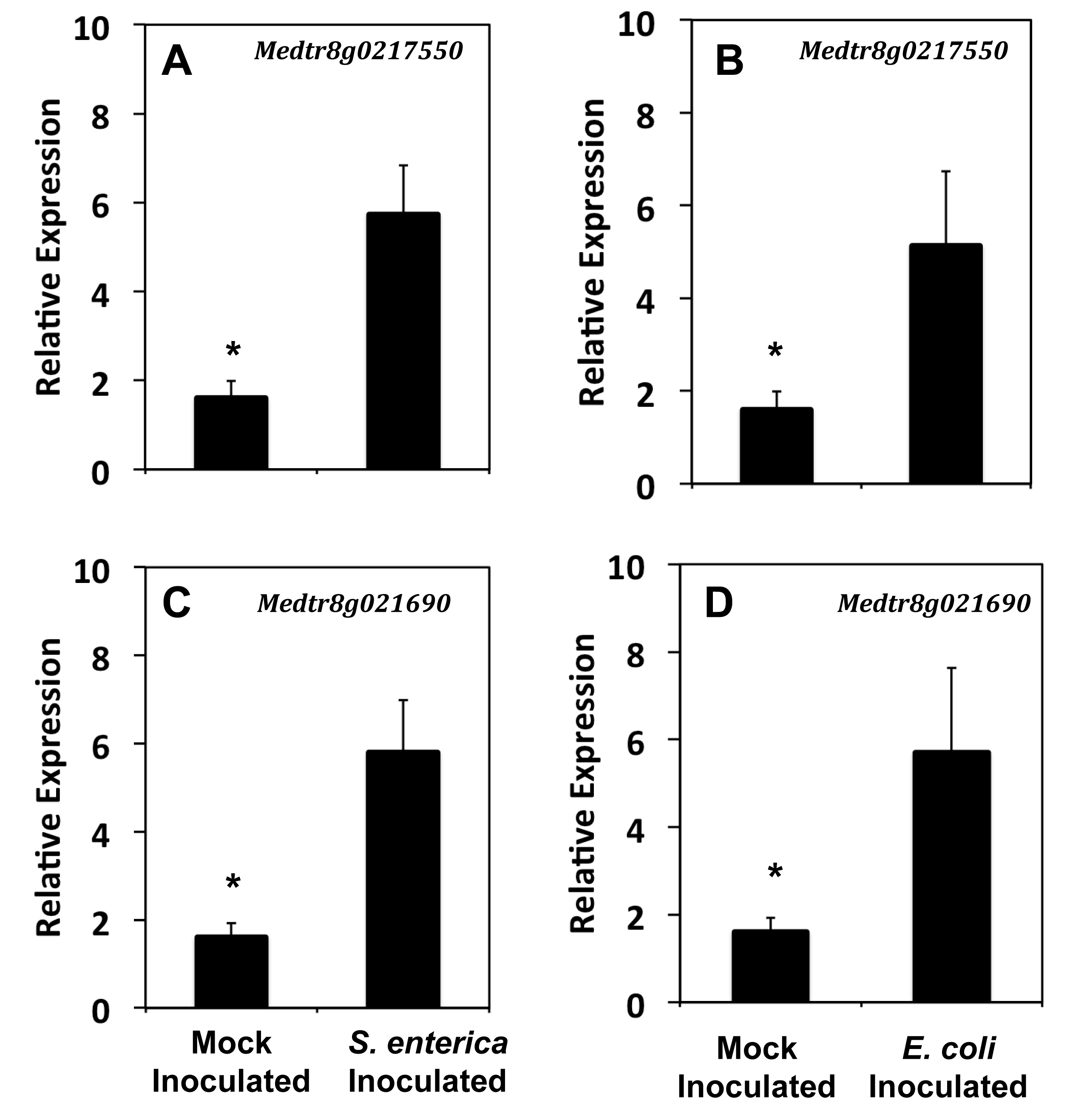

Supplement: Figure S2 — Validation of Medicago Affymetrix GeneChip® probe sets differentially expressed in response to inoculation with Salmonella enterica and E. coli O157:H7 cocktails by Quantitative RT-PCR. Expression analysis of 10 days post inoculation Medicago Jemalong A17 plants inoculated with 2 cfu/plant of either Salmonella (A and C) or E. coli O157:H7 (B and D) cocktail. Relative expression of lipooxygenases Medtr8g021750 and Medtr8g021690 in plants inoculated with Salmonella (A and C) and E. coli O157:H7 (B and D). Error bar represent the standard error of mean from three biological replicates. (TIF) [file pone.0087970.s002.tif]
