# Supplementary material for: Response of Medicago truncatula Seedlings to Colonization by Salmonella enterica and Escherichia coli O157:H7
Source: PLoS One. 2014 Feb 14;9(2):e87970. doi: 10.1371/journal.pone.0087970 (PMC3925098; doi:10.1371/journal.pone.0087970)
Supplement: Table S1 — List of bacterial serovars used for this study and the corresponding markers in their plasmid. (DOCX) [file pone.0087970.s003.docx]

**Supporting information Tables:**

**Table S1: List of bacterial serovars used for this study and the corresponding markers in their plasmid.**

| ***Serovar*** | **Host** | **Fluorescent**  **marker** |
| --- | --- | --- |
| *Salmonella enterica ssp. enterica ser. Schwarzengrund* | Alfalfa | GFP |
| *Salmonella enterica ssp. enterica ser. Enteritidis* | Tomato | GFP |
| *Salmonella enterica ssp. enterica ser. Mbandaka* | Seedlings | GFP |
| *Salmonella enterica ssp. enterica ser. Havana* | Seedlings | GFP |
| *Salmonella enterica ssp. enterica ser. Cubana* | Seedlings | GFP |
| *E. coli O157:H7 Odwalla* | Odwalla outbreak | *Ds*Red |
| *E. coli O157:H7 EDL933* | Spinach | GFP |
| *E. coli O157:H7 H2439* | Apple cider | GFP |
| *E. coli O157:H7 C7927* | Apple cider | GFP |
| *E. coli O157:H7 96A 13466* | Apple cider | GFP |
