# Supplementary material for: Response of Medicago truncatula Seedlings to Colonization by Salmonella enterica and Escherichia coli O157:H7
Source: PLoS One. 2014 Feb 14;9(2):e87970. doi: 10.1371/journal.pone.0087970 (PMC3925098; doi:10.1371/journal.pone.0087970)
Supplement: Table S2 — Probesets involved in biotic stress response with differential expression in Salmonella enterica and E.coli O157:H7 inoculated plants. The functional categories are in bold. IMGAG Annotation: Gene model predicted by International Medicago Genome Annotation Group (IMGAG) associated with the Probe set ID. Fold Change: Fold change expressed in Log2. A: Probesets involved in biotic stress response with differential expression in Salmonella enterica inoculated plants. The functional categories are in bold. IMGAG Annotation: Gene model predicted by International Medicago Genome Annotation Group (IMGAG) associated with the Probe set ID. Fold Change: Fold change expressed in Log2. B: Probesets involved in biotic stress response with differential expression in E.coli O157:H7 inoculated plants. The functional categories are in bold. IMGAG Annotation: Gene model predicted by International Medicago Genome Annotation Group (IMGAG) associated with the Probe set ID. Fold Change: Fold change expressed in Log2. (DOCX) [file pone.0087970.s004.docx]

**Table S2A: Probesets involved in biotic stress response with differential expression in *Salmonella* *enterica* inoculated plants**

| **Probe Set ID** | **IMGAG Annotation** | **Description** | **Fold Change** |
| --- | --- | --- | --- |
| ***a. Cell wall*** | | | |
| mtr.46426.1.s1_s_at | Medtr2g042560.1 | Cellulose synthase-like G1/2 (CSLG1/CSLG2/ATCSLG1) / transferase (transferring glycosyl groups) | +2.20 |
| mtr.50897.1.s1_at | Medtr4g081330.1 | Fasciclin-like arabinogalactan-protein 12 (FLA12) | +2.28 |
| mtr.18380.1.s1_at | Medtr4g081380.1 | Fasciclin-like arabinogalactan-protein 12 (FLA12) | +2.13 |
| mtr.37656.1.s1_at | Medtr3g016300.1 | Putative pectinesterase | +2.01 |
| ***b. Secondary metabolites*** | | | |
| mtr.40154.1.s1_at | Medtr5g098260.1 | 3-Hydroxy-3-methylglutaryl CoA reductase 1 (HMGR1/HMG1) | +2.05 |
| mtr.20438.1.s1_at | Medtr4g047310.1 Medtr4g047310.2 Medtr4g047310.3 | O-methyltransferase 1 (OMT1/ATOMT1) (lignin biosynthesis - COMT) | +3.20 |
| mtr.6500.1.s1_at | Medtr8g121370.1 | Putative mannitol dehydrogenase (lignin biosynthesis - CAD) | +2.01 |
| mtr.15281.1.s1_at | Medtr3g101260.1 | Transferase (flavonoids - anthocyanin 5-aromatic acyltransferase) | +2.29 |
| mtr.14428.1.s1_x_at | Medtr3g105280.1 | Chalcone synthase (CHS/TT4/ATCHS) | +2.32 |
| mtr.14428.1.s1_at | Medtr3g105280.1 | Chalcone synthase (CHS/TT4/ATCHS) | +2.15 |
| mtr.20567.1.s1_at | Medtr3g105290.1 | Chalcone synthase (CHS/TT4/ATCHS) | +2.41 |
| mtr.42370.1.s1_s_at | Medtr8g105350.1 Medtr8g105350.2 Medtr8g105360.2 | Oxidoreductase / 2OG-Fe(II) oxygenase family protein (flavonoids - flavonols) | +2.07 |
| ***c. PR proteins*** | | | |
| mtr.15817.1.s1_at | Medtr5g038650.1 | Putative disease resistance protein (TIR-NBS-LRR class) | +2.60 |
| mtr.34717.1.s1_at | Medtr3g040700.1 | Disease resistance-responsive family protein | +2.39 |
| mtr.45375.1.s1_at | Medtr7g076490.1 | Disease resistance-responsive protein-related / dirigent protein-related | +2.37 |
| ***d. Peroxidases*** | | | |
| mtr.40121.1.s1_at | Ac234842_16.1 | Peroxidase 22 (PER22/P22/PRXEA) | +2.16 |
| mtr.42373.1.s1_at | Medtr8g136930.1 | Peroxidase 40 (PER40/P40) | -2.53 |
| mtr.18570.1.s1_at | Medtr5g014310.1 | Peroxidase 20 (PER20/P20) | +2.06 |
| ***e. Signaling*** | | | |
| mtr.9308.1.s1_at | Medtr1g127100.1 | Putative photoassimilate-responsive protein (sugar and nutrient physiology) | +2.02 |
| mtr.10454.1.s1_at | Medtr7g111400.1 | Moderately similar to polygalactouronase inhibiting protein (PGIP1) (leucine-rich repeat [LRR] XI receptor kinase) | +2.75 |
| mtr.40240.1.s1_s_at | Medtr7g111400.1 | Moderately similar to polygalactouronase inhibiting protein (PGIP1) (LRR XI receptor kinase) | +2.53 |
| mtr.10055.1.s1_at | Medtr4g157930.1 | Putative protein kinase (DUF 26 receptor kinase) | +2.16 |
| mtr.29366.1.s1_at | Ac235009_57.1 Ac235006_17.1 | Highly similar to protein kinase family protein (DUF 26 receptor kinase) | +2.56 |
| mtr.51021.1.s1_s_at | Medtr4g157930.1 | Putative protein kinase (DUF 26 receptor kinase) | +2.69 |
| mtr.47587.1.s1_at | Medtr7g138010.1 Medtr7g138230.1 | Putative wall-associated receptor kinase-like 20 precursor | +2.64 |
| mtr.42339.1.s1_at | Medtr2g122150.1 | HPT phosphotransmitter 4 (AHP4) / histidine phosphotransfer kinase/transferase (phosphorelay) | +2.02 |
| ***f. Jasmonic acid*** | | | |
| mtr.24264.1.s1_at | Medtr8g021420.1 | LOX5 lipoxygenase (jasmonate synthesis - degradation) | +2.18 |
| mtr.50430.1.s1_at | Medtr8g021690.1 Medtr8g021690.2 | LOX5 lipoxygenase (jasmonate synthesis - degradation) | +6.29 |
| mtr.5628.1.s1_s_at | Medtr8g021750.1 Medtr8g021750.2 | LOX1 lipoxygenase (jasmonate synthesis - degradation) | +3.93 |
| mtr.46863.1.s1_s_at | Medtr8g021550.1 | LOX5 lipoxygenase (jasmonate synthesis - degradation) | +2.43 |
| mtr.50426.1.s1_at | Medtr8g021750.1 Medtr8g021750.2 | LOX1 lipoxygenase (jasmonate synthesis - degradation) | +4.46 |
| mtr.320.1.s1_x_at | Medtr8g006830.1 | 12-Oxophytodienoate reductase 2 (OPR2) | +2.65 |
| mtr.318.1.s1_at | Medtr5g006840.1 | 12-Oxophytodienoate reductase 2 (OPR2) | +3.19 |
| mtr.320.1.s1_at | Medtr5g006830.1 | 12-Oxophytodienoate reductase 2 (OPR2) | +2.49 |
| ***g. Ethylene*** | | | |
| mtr.44034.1.s1_at | Medtr2g050010.1 | Putative AP2 domain-containing transcription factor (ethylene-responsive element binding protein family) | -2.05 |
| mtr.14782.1.s1_at | Medtr3g122530.1 | Oxidoreductase / 2OG-Fe(II) oxygenase family protein (ethylene synthesis - degradation) | +2.15 |
| mtr.17787.1.s1_at | Medtr3g094020.1 | Oxidoreductase / 2OG-Fe(II) oxygenase family protein (ethylene synthesis - degradation) | +2.17 |
| mtr.46283.1.s1_s_at | Medtr2g088460.1 | 2-Oxoglutarate-dependent dioxygenase | +2.21 |
| mtr.38598.1.s1_at | Medtr4g128880.1 Medtr4g128770.1 | Oxidoreductase / 2OG-Fe(II) oxygenase family protein (ethylene synthesis - degradation) | +3.34 |
| ***h. Auxin*** | | | |
| mtr.12349.1.s1_at | Medtr7g140870.1 | Oxidoreductase (ATB2) (induced-regulated-responsive-activated) | +2.69 |
| mtr.166.1.s1_at | Medtr1g076620.1 | Auxin-responsive family protein (induced-regulated-responsive-activated) | -2.21 |
| ***i. Brassinosteroids*** | | | |
| mtr.45032.1.s1_at | Medtr4g122000.1 | Squalene expoxidase 1 (XF1) (oxidoreductase) | +2.25 |
| ***j. Redox*** | | | |
| mtr.12856.1.s1_at | Medtr7g129910.1 | Glutaredoxin family protein | +2.19 |
| ***k. Proteolysis*** | | | |
| mtr.11210.1.s1_at | Medtr3g157100.1 | Zinc finger (C3HC4-type RING finger) family protein (ubiquitin E3) | +2.19 |
| mtr.42240.1.s1_at | Medtr5g044570.1 | Zinc finger (C3HC4-type RING finger) family protein (ubiquitin E3) | -2.06 |
| ***l. Heat Shock*** | | | |
| mtr.50164.1.s1_at | Medtr5g088740.1 | Class I small heat shock protein (HSP17.6C-CI) | -2.14 |

**TableS2B: Probesets involved in biotic stress response with differential expression in *E.coli* O157:H7 inoculated plants**

| **Probe Set ID** | **IMGAG Annotation** | **Description** | **Log foldChange** |
| --- | --- | --- | --- |
| ***a. Cell Wall*** | | | |
| mtr.33908.1.s1_at | Medtr6g022510.1 | Pectate lyase family protein | +2.39 |
| mtr.19007.1.s1_at | Medtr4g111750.1 | Expansin 15 (EXP15) | +2.01 |
| mtr.8533.1.s1_s_at | Medtr5g044880.1 | Expansin 15 (EXP15) | +2.01 |
| mtr.31598.1.s1_at | Medtr5g011340.1 | Expansin 7 (EXP7) | +2.40 |
| mtr.50574.1.s1_at | Medtr7g010300.2 Medtr7g010300.1 | Expansin 5 (EXP5), Expansin 45 (EXP45) | +2.08 |
| ***b. Secondary metabolites*** | | | |
| mtr.40156.1.s1_at | Medtr5g026590.1 Medtr5g026600.1 Medtr5g026610.1 Medtr5g026620.1 | 3-Hydroxy-3-methylglutaryl CoA reductase 1 (HMGR1/HMG1) (isoprenoid/mevalonate pathway) | -2.00 |
| mtr.44529.1.s1_at | Medtr7g011310.1 | O-methyltransferase 1 (OMT1/ATOMT1) (Phenylpropanoids/lignin biosynthesis - COMT) | +3.80 |
| mtr.20438.1.s1_at | Medtr4g047310.1 Medtr4g047310.2 Medtr4g047310.3 | O-methyltransferase 1 (OMT1/ATOMT1) (Phenylpropanoids/lignin biosynthesis - COMT) | +2.38 |
| mtr.35413.1.s1_at | Medtr7g011150.1 | O-methyltransferase 1 (OMT1/ATOMT1) Phenylpropanoids/lignin biosynthesis - COMT) | +2.39 |
| mtr.14428.1.s1_x_at | Medtr3g105280.1 | Chalcone synthase (CHS/TT4/ATCHS) (flavonoids - chalcones) | +2.36 |
| mtr.40122.1.s1_s_at | Medtr5g007860.1 | Chalcone synthase (CHS/TT4/ATCHS) (flavonoids - chalcones) | +2.87 |
| mtr.14428.1.s1_at | Medtr3g105280.1 | Chalcone synthase (CHS/TT4/ATCHS) (flavonoids - chalcones) | +2.29 |
| mtr.17616.1.s1_x_at | Medtr7g014960.1 | Chalcone synthase (CHS/TT4/ATCHS) (flavonoids - chalcones) | +2.68 |
| mtr.20567.1.s1_at | Medtr3g105290.1 | Chalcone synthase (CHS/TT4/ATCHS) (flavonoids - chalcones) | +2.90 |
| mtr.20464.1.s1_x_at | Medtr7g014940.1 | Chalcone synthase (CHS/TT4/ATCHS) (flavonoids - chalcones) | +3.01 |
| mtr.42370.1.s1_s_at | Medtr8g105350.1 Medtr8g105350.2 Medtr8g105360.2 | Oxidoreductase 2OG-Fe(II) oxygenase family protein (flavonoids - flavonols) | +2.31 |
| mtr.24231.1.s1_at | Medtr4g095930.1 | Putative isoflavone reductase (flavonoids - isoflavonols) | +2.37 |
| mtr.24228.1.s1_at | Medtr4g095900.1 Medtr4g095900.2 | Putative isoflavone reductase (flavonoids - isoflavonols) | +2.08 |
| ***c. PR proteins*** | | | |
| mtr.16372.1.s1_at | Medtr6g073710.1 | Putative disease resistance protein (TIR-NBS-LRR class) | +2.45 |
| mtr.34717.1.s1_at | Medtr3g040700.1 | Disease resistance-responsive family protein | +2.47 |
| mtr.16372.1.s1_x_at | Medtr6g073710.1 | Putative disease resistance protein (TIR-NBS-LRR class) | +2.17 |
| ***d. Peroxidase*** | | | |
| mtr.15379.1.s1_at | Medtr8g129440.1 | Putative peroxidase | +2.06 |
| msa.2680.1.s1_at | Medtr5g091290.1 | Putative peroxidase | +2.05 |
| mtr.40121.1.s1_at | Ac234842_16.1 | Peroxidase 22 (PER22/P22/PRXEA) / basic peroxidase E | +3.07 |
| mtr.42373.1.s1_at | Medtr8g136930.1 | Peroxidase 40 (PER40/P40) | -2.33 |
| mtr.14635.1.s1_at | Medtr5g082680.1 | Putative peroxidase | +2.48 |
| mtr.18570.1.s1_at | Medtr5g014310.1 | Peroxidase 20 (PER20/P20) | +2.06 |
| ***e. Signaling*** | | | |
| mtr.9308.1.s1_at | Medtr1g127100.1 | Putative photoassimilate-responsive protein (sugar and nutrient physiology) | +2.81 |
| mtr.10454.1.s1_at | Medtr7g111400.1 | Polygalacturonase inhibiting protein 1 (PGIP1) (leucine-rich repeat [LRR] XI receptor kinase) | +2.13 |
| mtr.22569.1.s1_s_at | Medtr2g018130.1 Medtr5g105480.1 Medtr5g102650.1 Medtr1g046810.1 | Haesa-Like 1 (HSL1) / ATP binding / serine/threonine kinase (LRR XI receptor kinase) | -2.07 |
| mtr.21812.1.s1_at | Medtr5g034280.1 | Leucine-rich repeat family protein (LRR XI receptor kinase) | +2.43 |
| mtr.21812.1.s1_s_at | Medtr5g034280.1 | Leucine-rich repeat family protein (LRR XI receptor kinase) | +2.02 |
| mtr.14277.1.s1_s_at | Ac202489_12.1 Medtr5g076990.1 | Protein kinase family protein / cysteine-rich receptor-like protein kinase 36 precursor (LRR XI receptor kinase) | -2.13 |
| mtr.38541.1.s1_at | Ac235005_26.1 | Protein kinase family protein (DUF 26 receptor kinase) | +2.15 |
| mtr.45715.1.s1_at | Medtr2g041370.1 | Lectin protein kinase family protein (DUF 26 receptor kinase) | +2.23 |
| mtr.21729.1.s1_at | Medtr8g044960.1 | Receptor-like kinase 6 (RLK6) / Cysteine-rich RLK 5 (CRK5) (DUF 26 receptor kinase) | +2.65 |
| mtr.50502.1.s1_at | Medtr8g045080.1 | S-locus lectin protein kinase family protein (DUF 26 receptor kinase) | +2.06 |
| mtr.20739.1.s1_s_at | Medtr2g041340.1 | Protein kinase family protein (DUF 26 receptor kinase) | +2.05 |
| ***f. Jasmonic acid*** | | | |
| mtr.50430.1.s1_at | Medtr8g021690.1 Medtr8g021690.2 | LOX5 lipoxygenase (jasmonate synthesis - degradation) | +4.61 |
| mtr.5628.1.s1_s_at | Medtr8g021750.1 Medtr8g021750.2 | LOX1 lipoxygenase (jasmonate synthesis - degradation) | +3.38 |
| mtr.46863.1.s1_s_at | Medtr8g021550.1 | LOX5 lipoxygenase (jasmonate synthesis - degradation) | +2.13 |
| mtr.50426.1.s1_at | Medtr8g021750.1 Medtr8g021750.2 | LOX1 lipoxygenase (jasmonate synthesis - degradation) | +3.93 |
| ***g. Ethylene*** | | | |
| mtr.22007.1.s1_at | Medtr8g008670.1 | Putative 2-oxoglutarate-dependent dioxygenase (ethylene synthesis - degradation) | +2.00 |
| mtr.46283.1.s1_s_at | Medtr2g088460.1 | Putative 2-oxoglutarate-dependent dioxygenase (ethylene synthesis - degradation) | +2.74 |
| ***h. WRKY*** | | | |
| mtr.48109.1.s1_at | Medtr3g138240.1 | WRKY DNA-binding protein 40 (ATWRKY40/WRKY40) / transcription factor | +2.06 |
| ***i. Auxin*** | | | |
| mtr.12349.1.s1_at | Medtr7g140870.1 | Oxidoreductase (ATB2) (induced-regulated-responsive-activated) | +2.18 |
