# Supplementary material for: Response of Medicago truncatula Seedlings to Colonization by Salmonella enterica and Escherichia coli O157:H7
Source: PLoS One. 2014 Feb 14;9(2):e87970. doi: 10.1371/journal.pone.0087970 (PMC3925098; doi:10.1371/journal.pone.0087970)
Supplement: Table S3 — List of primers used for Quantitative RT-PCR. (DOCX) [file pone.0087970.s005.docx]

**Table S3: List of primers used for Quantitative RT-PCR.**

| **Primer name** | **Sequence from 5′ to 3′** |
| --- | --- |
| Medtr2g036460F | CACTATCAGTTTCCTGATTCTCCTCCTTCC |
| Medtr2g036460R | TATTACCACCACTCCATATACAGATTCTGC |
| Medtr8g021750F | GCAGCAGTTAACTTTGGACAGTATCC |
| Medtr8g021750R | CTGGTCCAGTGCGGTTTTTCAAACTAG |
| Medtr8g021690F | GGTGACTTAAAGAGTGCCACATGGT |
| Medtr8g021690R | GGTCTGTAAGGGTATCACTCTTGGG |
| *EF1a*F | GTCAAAACATGGTTGCTGCACAAGC |
| *EF1a*R | TTAGGTCACAAGGCAGATTGCAGG |
| Mt*ACTIN*F | GCAGATGCTGAGGATATTAACCC |
| Mt*ACTIN*R | CGACCACTTGCATAGAGGGAGAGG |
